# Editorial: Minimally Invasive Repair of Ventral and Incisional Hernias

**DOI:** 10.3389/jaws.2025.15292

**Published:** 2025-08-05

**Authors:** Dimitrios Prassas

**Affiliations:** ^1^ Heinrich Heine University of Düsseldorf and Düsseldorf University Hospital, Department of Surgery (A), Düsseldorf, Germany; ^2^ Department of General and Visceral Surgery, Katholisches Klinikum Essen, Essen, Germany

**Keywords:** minimally invasive surgery, ventral hernia, trans-abdominal preperitoneal (TAPP), extended view totally extraperitoneal repair (eTEP), intraperitoneal onlay mesh technique (IPOM)

Ventral and incisional hernias are a particularly common occurrence, impacting millions of patients worldwide. The biomechanical complexity of the abdominal wall serves both as the foundation for understanding its pathologies and as a gateway to a range of therapeutic strategies. One of the very first applications of minimally invasive surgery in the early nineties was that of laparoscopic repair of abdominal wall hernias with the intraperitoneal onlay mesh (IPOM) technique. Today, over three decades later, various minimally invasive approaches have been introduced, with the question of the most appropriate one still remaining open. Three out of nine papers included in the present special issue compared the outcome of IPOM to that of other approaches.

Anusitviwat et al. conducted a head-to-head comparison of laparoscopic eTEP-RS/TAR and IPOM techniques, highlighting the advantages of the former in cases with medium- to large-sized hernias. Munjuluri et al. indicated in their cohort that cases treated with laparoscopic ventral TAPP demonstrated lower postoperative pain and reduced costs compared to laparoscopic IPOM plus. Finally, Singh et al. conducted a systematic review and meta-analysis of randomized controlled trials examining the impact of the robotic platform on the outcome of IPOM when compared to the laparoscopic approach, without noting any differences other than higher operational costs for those treated robotically.

Furthermore, a second systematic review and meta-analysis was included, analyzing three studies comparing robot-assisted enhanced-view totally extraperitoneal (eTEP) and transabdominal retromuscular (TARM, aka TARUP) ventral hernia mesh repair. Brucchi et al. did not find any striking differences between the two methods but underlined the need for further trials examining these technically similar techniques and unmasking subtle differences, if any exist. Radu share with us their approach regarding robotic PeTEP for a case with incisional hernia and, in a second paper, demonstrate their personal experience from the first five years conducting eTEP/eTEP-TAR repairs. Vogel et al. share their experience from their first 160 consecutive robotically assisted lateral eTEP and eTAR techniques, providing us with interesting insights regarding that matter. Van Hoef et al. contributed an interesting work focusing on repair of lateral abdominal wall defects with open or robotic unilateral transversus abdominis release, showing a shorter length of stay using the robotic approach in the short-term follow-up.

Last but not least, the rapid rise of Artificial Intelligence and its implications in abdominal wall reconstruction were explored in the final paper of our special issue by Vogel and Mück, shedding light on the rather bright future that lies ahead.

The goal of this Special Issue is to provide a comprehensive overview of the already established, as well as emerging, minimally invasive techniques for ventral and incisional hernia repair with regard to their outcome, as an attempt to determine the most appropriate indications for each approach in a given setting. I would like to personally thank all 32 authors for their thorough work and dedication to this fascinating, ever-evolving field of abdominal wall surgery.

